# Exploring associations between parental and peer variables, personal variables and physical activity among adolescents: a mediation analysis

**DOI:** 10.1186/1471-2458-14-966

**Published:** 2014-09-18

**Authors:** Maïté Verloigne, Jenny Veitch, Alison Carver, Jo Salmon, Greet Cardon, Ilse De Bourdeaudhuij, Anna Timperio

**Affiliations:** Department of Movement and Sport Sciences, Ghent University, Ghent, Belgium; Centre for Physical Activity and Nutrition Research (C-PAN), Deakin University, Melbourne, Australia

**Keywords:** Physical activity, Adolescent, Parents, Peers, Self-efficacy, Barriers, Accelerometer

## Abstract

**Background:**

This study aimed to investigate how parental and peer variables are associated with moderate- to-vigorous intensity physical activity (MVPA) on week- and weekend days among Australian adolescents (13-15y), and whether perceived internal barriers (e.g. lack of time), external barriers (e.g. lack of others to be physically active with) and self-efficacy mediated these associations.

**Methods:**

Cross-sectional data were drawn from the Health, Eating and Play Study, conducted in Melbourne, Australia. Adolescents (mean age = 14.11 ± 0.59 years, 51% girls) and one of their parents completed a questionnaire and adolescents wore an ActiGraph accelerometer for a week (n = 134). Mediating effects of perceived barriers and self-efficacy were tested using MacKinnon’s product-of-coefficients test based on multilevel linear regression analyses.

**Results:**

Parental logistic support was positively related to MVPA on weekdays (τ = 0.035) and weekend days (τ = 0.078), peer interest (τ =0.036) was positively related to MVPA on weekdays, and parental control (τ = −0.056) and parental concern (τ = −0.180) were inversely related to MVPA on weekdays. Internal barriers significantly mediated the association between parental logistic support and MVPA on weekdays (42.9% proportion mediated). Self-efficacy and external barriers did not mediate any association.

**Conclusions:**

Interventions aiming to increase adolescents’ MVPA should involve parents, as parental support may influence MVPA on weekdays by reducing adolescents’ perceived internal barriers. Longitudinal and experimental research is needed to confirm these findings and to investigate other personal mediators.

## Background

Physical activity (PA) is important for adolescents’ physical and mental health, and is acknowledged as an important target in obesity prevention [1, 2]. Accordingly, it is recommended for adolescents to participate in at least 60 minutes of moderate-to-vigorous PA (MVPA) per day [2]. However, more than 75% of adolescents fail to meet MVPA guidelines [3, 4], suggesting the need for continuous efforts to increase adolescents’ MVPA. To develop effective interventions, an essential step is to identify factors associated with adolescents’ MVPA. This step requires the application and evaluation of a theoretical approach to gain a clear understanding of factors related to health behaviour [5]. The socio-ecological model [6] and the Environmental Research framework for weight Gain prevention or the EnRG framework [7] both integrate personal factors with environmental factors to understand why individuals perform (un)healthy behaviours.

There is evidence that both parents and peers influence adolescents’ PA [8–10]. Parents have a strong influence on their child’s behaviour from early childhood and continue to be influential when their children reach adolescence; however the influence of peers is believed to increase with age [11]. There are several means through which parents and peers can have an impact on adolescents’ PA, such as through encouragement [12, 13], logistic support [13], and modeling [14–16]. Thus, it is important to consider the role of both parents and peers as influences on adolescents’ PA.

Further, according to the socio-ecological model [6] and the EnRG framework [7], these parental and peer variables can directly influence health behaviour via ‘automatic’ pathways when people spontaneously engage in a particular behaviour or indirectly via adolescents’ personal or individual-level factors. Investigating the mediating effect of personal factors on how parents and peers influence PA enables an in-depth insight into the process that may underlie adolescents’ behaviour and has been receiving growing attention in recent literature [5]. Two important personal or individual-level factors are perceived barriers to PA and self-efficacy [17, 18] which may be developed and/or reinforced by parents and peers [19].

Self-efficacy represents a belief in one’s capability to engage in PA under challenging conditions or situations, and has been identified as a strong correlate of adolescents’ PA [20, 21]. Several studies have demonstrated a mediating effect of self-efficacy on associations between the family environment and the consumption of sugar-sweetened beverages [22] and fruit [23], and between the perceived physical environment and PA [8, 24] among adolescents. Perceived barriers to PA (e.g. lack of time because of homework and other scheduled activities, lack of interest in PA) have been shown to be related to adolescents’ PA [20], but have received less attention as potential mediators of associations between environmental factors and PA. Only one previous study has identified perceived barriers as a mediator of the association between parental social support and PA among US adolescent girls [18]. Thus, limited research has examined the mediating role of self-efficacy or perceived barriers on associations between parental or peer variables and PA. Investigating potential mediators will inform future interventions aimed at promoting adolescents’ MVPA on the potential mechanisms of behaviour change.

Therefore, the aim of this study was to examine associations between parental and peer variables and MVPA on week- and weekend days, and the potential mediating effect of perceived barriers (e.g. lack of time, lack of others to be physically active with,…) and self-efficacy on these associations among Australian adolescents, aged 13–15 years. Given that adolescents’ MVPA levels differ between week- and weekend days, factors related to MVPA may also differ [25, 26].

## Methods

### Procedure

Cross-sectional data presented in this paper were drawn from the first follow-up (2006, T2) of the older cohort within the Health, Eating and Play Study (HEAPS), as the focus of this paper was on adolescents [27–29]. At baseline in 2002/2003 (T1), stratified random sampling proportionate to school size (enrolments > 200) in low, middle and high socio-economic status areas were used to select elementary schools from greater Melbourne, Australia. In total, 24 of the 39 elementary schools approached agreed to participate. All children in Grade Prep (i.e. first year of elementary school) were invited to participate (younger cohort). In 17 of the 24 schools, children in the 5th and 6th grade were also invited to participate (older cohort). In total, 947 older children (mean age = 11.2y) participated in T1 and parents of 474 of these children agreed to be re-contacted for further follow-up measures. At T2, those older children (now aged 13-15y) and their parents were invited to participate in a follow-up of which 200 provided written parental consent (42%). An adolescent and parental questionnaire was mailed to participants and adolescents were also asked to wear an accelerometer for eight consecutive days (167 agreed). In total, complete adolescent and parental questionnaire and accelerometer data were collected from 134 adolescents (67%) at T2. Those who were followed up had a higher level of maternal education (45% vs. 33% high education, p < 0.05) and weekend MVPA (65.1 vs. 55.6 mins/day, p < 0.05) than the remainder of the baseline sample. Approval was provided by the Deakin University Human Research Ethics Committee, the Victorian Department of Education and Training and the Catholic Education Office.

### Measures

**Demographic variables.** The parental questionnaire assessed highest level of maternal education and parental age. Maternal education was collapsed into three categories: low (did not complete high school), medium (high school or technical or trade certificate) or high (University or tertiary qualification). The adolescent questionnaire assessed adolescents’ age and sex.

**Parental and peer variables.** Five parental constructs were assessed in the parent questionnaire: (a) parental co-participation in PA, (b) parental logistic support for PA (included two items; Cronbach’s alpha = 0.90), (c) parental praise of PA, (d) parental control of PA (included four items; Cronbach’s alpha = 0.46), and (e) parental concern about PA (Table 1). Four peer constructs were assessed in the adolescent questionnaire: (a) peer modeling of PA, (b) peer enjoyment of PA, (c) peer interest in PA, and (d) peer encouragement of PA (Table 1). Parental co-participation, parental logistic support, parental praise and all peer constructs were adapted from a reliable parent and peer support scale [30], that was already used in previous studies [30, 31]. Parental control and parental concern were adapted from the Child Feeding Questionnaire [32] to be relevant in the PA domain.

**Personal variables.** Personal variables were assessed in the adolescent questionnaire (Table 1). The mean value of nine items was used to measure self-efficacy (Cronbach’s alpha = 0.84). These items were adapted from the Self-Efficacy scale that was based on the Social Cognitive Theory [33] and was used in a questionnaire to measure psychosocial determinants of adolescents’ behaviour [34]. A higher mean value represents more self-efficacy to overcome barriers. Twelve items assessed adolescents’ perceived barriers to PA. These items were adapted to the adolescent population from the Pilot Survey of the fitness of Australians [35]. Exploratory factor analyses revealed two factors, with a total variance explained of 52.5%. Seven items loaded on the first factor which could be labeled as the perception of ‘internal barriers’ (Cronbach’s alpha = 0.87). Four items loaded on the second factor which could be labeled as the perception of ‘external barriers’ (Cronbach’s alpha = 0.66). One item (‘I look funny when I am doing physical activity’) did not load on either factor and was therefore not included in further analyses. The mean values of responses to items within the two factors were calculated with higher mean values representing a stronger perception of internal or external barriers.

**Table 1:** **Description of parental, peer and personal variables**

Parental variables	Item (Parent questionnaire)	Response category
Parental co-participation in PA; adapted from [31]	Thinking about the past few months, how often did you and/or the co-carer do the following things together with your child?	1 = Never or rarely; 2 = Less than once/week; 3 = Once a week; 4 = About 2–3 times/week; 5 = About 4–6 times/week; 6 = Every day
	- I/we did physical activity, sport or exercise together with the child	
Parental logistic support for PA; adapted from [31]	Mean value of two items (Cronbach’s alpha = 0.90):	1 = Never or rarely; 2 = Less than once/week; 3 = Once a week; 4 = About 2–3 times/week; 5 = About 4–6 times/week; 6 = Every day; 7 = Doesn’t apply^a^
	Thinking about the past few months, how often were the following true?	
	- The co-carer or I took the child to sports training/lessons	
	- The child did sport or physical activity that the co-carer or I provided money for	
Parental praise of PA; adapted from [31]	Thinking about the past few months, how often was the following true?	1 = Never or rarely; 2 = Less than once/week; 3 = Once a week; 4 = About 2–3 times/week; 5 = About 4–6 times/week; 6 = Every day; 7 = Doesn’t apply^a^
	- The co-carer or I praised the child participating in physical activity, sport or exercise	
Parental control of PA; adapted from [32]	Mean value of four items (Cronbach’s alpha = 0.46):	1 = Disagree; 2 = Slightly disagree; 3 = Neutral; 4 = Slightly agree; 5 = Agree
	Indicate how much you agree with following statements.	
	- If I did not guide or regulate my child’s activities, (s)he would not be as active as (s)he should be	
	- I think that my child should be active every day	
	- I am careful to make sure that my child gets enough exercise	
	- If my child says “I’m tired”, I try to get him/her to exercise anyway	
Parental concern about PA; adapted from [32]	How concerned are you about your child not getting enough physical activity?	1 = Not concerned; 2 = A little concerned; 3 = Fairly concerned; 4 = Very concerned
**Peer variables; adapted from** [31]	**Item (Adolescent questionnaire)**	**Response category**
Peer modeling of PA	My closest friend does a lot of physical activity	1 = Strongly agree; 2 = Agree; 3 = Neither; 4 = Disagree; 5 = Strongly disagree; 6 = Don’t know^b^
Peer enjoyment of PA	My closest friend enjoys physical activity or sports	1 = Strongly agree; 2 = Agree; 3 = Neither; 4 = Disagree; 5 = Strongly disagree; 6 = Don’t know^b^
Peer interest in PA	My closest friend is not the sporty type	1 = Strongly agree; 2 = Agree; 3 = Neither; 4 = Disagree; 5 = Strongly disagree; 6 = Don’t know^b^
Peer encouragement of PA	Mean value of three items (Cronbach’s alpha = 0.70):	1 = Never/rarely; 2 = Sometimes; 3 = Often
	During the past year, how often have your friends said or done this:	
	- Encouraged me to do more physical activity	
	- Encouraged me to walk to or from school or to other places	
	- Encouraged me to play sports	
**Personal variables**	**Item (Adolescent questionnaire)**	**Response category**
Self-efficacy to overcome barriers; adapted from [34]	Mean value of nine items (Cronbach’s alpha =0.84):	1 = Not at all sure; 2 = A bit sure; 3 = Fairly sure; 4 = Quite sure; 5 = Very sure
	I could be active even…	
	- If there is no one to be active with	
	- If I don’t have enough energy to be active	
	- If I am not good at it	
	- If I had no one to take me training	
	- If my friends don’t take part	
	- If the weather is bad	
	- If I had a lot of homework to do	
	- If I were busy going out with my friends	
	- If others make fun of me	
Perceived internal barriers; adapted from [35]	Mean value of seven items (Cronbach’s alpha = 0.87):	1 = Strongly agree; 2 = Agree; 3 = Neither; 4 = Disagree; 5 = Strongly disagree; 6 = Don’t know^b^
	How much do you agree with the following statements?	
	- I don’t have enough time for physical activity	
	- I prefer to watch TV or play electronic games	
	- I don’t like physical activity	
	- I don’t think I’m very good at physical activity	
	- I don’t like how being active physically makes me feel (e.g. hot, sweaty, out of breath)	
	- I’m not the sporty type	
	- I am too lazy/ can’t be bothered	
Perceived external barriers; adapted from [35]	Mean value of four items (Cronbach’s alpha = 0.66):	1 = Strongly agree; 2 = Agree; 3 = Neither; 4 = Disagree; 5 = Strongly disagree; 6 = Don’t know^b^
	How much do you agree with the following statements?	
	- I don’t have anyone to be physically active with	
	- I have a health problem or injury that prevents me from being physically active	
	- I can’t afford to buy sports clothes or equipment, or pay sport/club fees	
	- I think it’s too dark and cold in winter to spend more time outside	

^a^Response possibility 7 (Doesn’t apply) was re-coded into 1 (Never/rarely); ^b^response possibility 6 (Don’t know) was re-coded into 3 (Neither).

**Moderate-to-vigorous intensity physical activity.** ActiGraph uniaxial accelerometers (model 7162) were used to objectively measure adolescents’ MVPA on weekdays and weekend days, as these instruments have acceptable reliability and validity for use in this study population [36, 37]. Adolescents were instructed to wear the accelerometer for all waking hours, but to remove it during water-based activities. Selected epoch length was 60s and non-wear time was defined as periods of >20mins of consecutive zero counts. Adolescents with at least three valid weekdays (MVPA weekday) and one valid weekend day (MVPA weekend day), defined as a minimum of 8 h wear-time, were included in analyses. The age-specific cut-points of Freedson [38] were used to estimate the time spent per day in moderate (4.0-5.9 Metabolic Equivalent of Tasks or METS) and vigorous (≥6 METS) PA [39]. Taking the age of a child into account when estimating moderate and vigorous PA is important because of the variation in oxygen consumption at rest and during submaximal exercise [40]. When using age-specific cut-points, older children in the sample have to reach a higher number of accelerometer counts to reach the threshold of 4 METS. Minutes per weekday and weekend day in MVPA were calculated by summing and averaging these values across valid days.

### Statistical analyses

Linear regression analyses were conducted using SPSS version 22.0 (SPSS Inc., Chicago, IL, USA). Clustering at the school level was taken into account by conducting multi-level analyses. Skewed variables (MVPA on weekdays and weekend days, perceived internal/external barriers, parental concern, peer enjoyment, peer encouragement) were log-transformed to improve distributions. Because of their potential relationship with MVPA, maternal education and adolescents’ sex were included as covariates in all analyses. Total accelerometer wear-time and days worn were included a-priori in all analyses involving MVPA outcomes.

**Figure 1:**
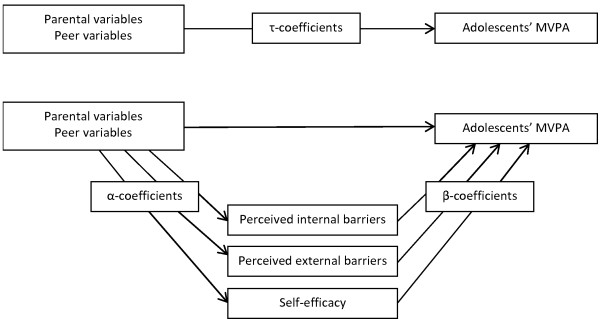
**Mediation model of the association between parental and peer variables, personal variables (perceived internal and external barriers and self-efficacy) and adolescents’ MVPA.**

The mediation analyses that are presented in Figure 1, consisted of the following steps. Firstly, main associations between each parental or peer variable and adolescents’ MVPA on weekdays and weekend days were examined (τ-coefficient). In the Results, we report the magnitude of the significant associations based on back-transformation of the log-transformed τ-coefficient. In the second stage, the mediating role of perceived internal/external barriers and self-efficacy was examined using the product-of-coefficients test of MacKinnon et al. [41]. This test includes the following steps: (1) estimation of the associations between each parental/peer variable and potential mediators (Action Theory test; α-coefficients or a-path); (2) estimation of the associations between the potential mediators and adolescents’ MVPA on weekdays and weekend days (Conceptual Theory Test; β-coefficients or b-path), adjusting for the relevant parental/peer variable; and (3) calculation of the product-of-coefficients (αβ), representing the mediated effect. Statistical significance of the mediated effect was estimated by dividing αβ by its standard error (SE). To calculate SE, the Sobel test was used: SE (αβ) = √(α^2^*SE (β)^2^ + β^2^*SE (α)^2^) [42]. The percentage mediating the association between parental/peer variables and adolescents’ MVPA on weekdays and weekend days was calculated by dividing αβ by the τ-coefficient. Due to the small sample size, results close to significance (0.1 level) were indicated, along with significance at the p < 0.05 level.

## Results

Table 2 provides an overview of sample characteristics and descriptive statistics of variables.

**Table 2:** **Sample characteristics and descriptive statistics (n = 134)**

Demographic variables	% or mean ± SD
Sex, girls (%)	51
Adolescent’s age (mean ± SD, years)	14.11 ± 0.59
Maternal education (%)	
- Low	18
- Medium	41
- High	41
Person who completed parent questionnaire (%)	
- Mother/female carer	84
- Father/male carer	15
- Other	1
Age of responding parent (mean ± SD, years)	45.02 ± 4.72
**PA variables**	
MVPA on weekdays (mins/day)	45.49 ± 23.65
MVPA on weekend days (mins/day)	30.80 ± 29.63
**Parental variables**	
Parental co-participation in PA (range 1–6)	2.27 ± 1.12
Parental logistic support for PA (range 1–6)	3.25 ± 1.34
Parental praise of PA (range 1–6)	3.49 ± 1.44
Parental control of PA (range 1–5)	3.23 ± 0.74
Parental concern about PA (range 1–4)	2.04 ± 1.42
**Peer variables**	
Peer modeling of PA (range 1–5)	2.48 ± 1.14
Peer enjoyment of PA (range 1–5)	2.11 ± 0.97
Peer interest in PA (range 1–5)	3.62 ± 1.23
Peer encouragement of PA (range 1–3)	1.41 ± 0.49
**Personal variables**	
Self-efficacy to overcome barriers (range 1–5)	3.40 ± 0.81
Perceived internal barriers (range 1–5)	1.88 ± 0.67
Perceived external barriers (range 1–5)	1.85 ± 0.64

SD, standard error; MVPA, moderate- to vigorous physical activity; mins, minutes.

### Associations between parental and peer variables and MVPA (τ-coefficient)

Main effects for associations between parental/peer variables and MVPA are presented in Table 3. For MVPA on weekdays, a significant positive association was found with parental logistic support and peer interest. For each one unit increase in parental logistic support and peer interest, mean MVPA changed by a factor of 1.08 and 1.09, respectively. A significant inverse association was found with parental control and concern. For each one unit increase in parental control and concern, mean MVPA changed by a factor of 0.88 and 0.66, respectively. For MVPA on weekend days, a significant positive association was found with parental logistic support. For each one unit increase in parental logistic support, mean MVPA changed by a factor of 1.20. Associations with parental praise and co-participation were close to significant and in a positive direction. For each one unit increase in parental praise and co-participation, mean MVPA changed by a factor of 1.15 and 1.19, respectively.

**Table 3:** **Main associations between parental and peer variables and MVPA on weekdays and weekend days (mins/day)**

	MVPA on weekdays	MVPA on weekend days
	τ, SE (95% CI)	τ, SE (95% CI)
**Parental variables**		
Parental co-participation in PA	0.025, 0.019	*0.076, 0.041*
	(−0.012;0.062)	*(−0.004;0.156)* ^†^
Parental logistic support for PA	**0.035, 0.016**	**0.078, 0.035**
	(0.004;0.066)*	(0.009;0.147)*
Parental praise of PA	0.015, 0.014	*0.061, 0.032*
	(−0.012;0.042)	*(−0.002;0.124)* ^†^
Parental control of PA	**−0.056, 0.027**	−0.017, 0.063
	(−0.109;-0.003)*	(−0.140;0.106)
Parental concern about PA	**−0.180, 0.081**	−0.075, 0.181
	(−0.339;-0.021)*	(−0.430;0.280)
**Peer variables**		
Peer modeling of PA	−0.016, 0.019	−0.011, 0.042
	(−0.053;0.021)	(−0.096;0.068)
Peer enjoyment of PA	−0.131, 0.113	0.039, 0.244
	(−0.352;0.090)	(−0.439;0.517)
Peer interest in PA	**0.036, 0.018**	0.052, 0.039
	(0.001;0.071)*	(−0.024;0.128)
Peer encouragement of PA	0.086, 0.157	−0.342, 0.356
	(−0.222;0.394)	(−1.040;0.356)

Analyses were adjusted for adolescents’ sex, maternal education, total accelerometer wear-time and days worn; MVPA, moderate- to vigorous physical activity; SE, standard error; CI, confidence interval; ^**†**^p < 0.1 (indicated in italic); *p < 0.05 (indicated in bold).

### Associations between parental and peer variables and potential mediators (α-coefficients)

With the exception of parental co-participation, the majority of family/peer variables examined in this study were associated with the three potential mediators (p < 0.05; Table 4). Higher parental logistic support, peer interest in PA and parental praise were related to higher self-efficacy and the perception of less internal and external barriers. In contrast, higher parental control and parental concern were related to lower self-efficacy and the perception of more internal barriers. Higher parental concern was also related to the perception of more external barriers.

**Table 4:** **Mediation analyses for associations between parental and peer variables and MVPA on weekdays and weekend days**

MVPA on weekdays				
	**α, SE**	**β, SE**	**αβ, SE**	**% mediated**
	**(95%CI)**	**(95%CI)**	**(95%CI)**	
**Parental logistic support for PA**				
*Potential mediators*				
Self-efficacy	**0.175, 0.054**	0.032, 0.028	0.006, 0.005	-
	(0.069;0.281)**	(−0.023;0.087)	(−0.005;0.016)	
Internal barriers	**−0.044, 0.010**	**−0.341, 0.153**	**0.015, 0.008**	42.9%
	(−0.064;-0.024)***	(−0.641;-0.041)*	(0.000;0.030)*	
External barriers	**−0.040, 0.009**	−0.249, 0.157	0.010, 0.007	-
	(−0.058;-0.022)***	(−0.557;0.059)	(−0.003;0.023)	
**Parental control of PA**				
*Potential mediators*				
Self-efficacy	**−0.367, 0.099**	0.027, 0.028	−0.010, 0.011	-
	(−0.561;-0.173)***	(−0.028;0.082)	(−0.031;0.011)	
Internal barriers	**0.054, 0.018**	**−0.301, 0.145**	*−0.016, 0.010*	29.0%
	(0.019;0.089)**	(−0.585;-0.017)*	*(−0.035;0.002)* ^†^	
External barriers	0.020, 0.018	*−0.284, 0.149*	−0.006, 0.006	-
	(−0.015;0.055)	*(−0.576;0.008)* ^†^	(−0.017;0.006)	
**Parental concern about PA**				
*Potential mediators*				
Self-efficacy	**−1.099,0.291**	0.023, 0.028	−0.025, 0.031	-
	(−1.669;-0.529)***	(−0.032;0.078)	(−0.087;0.036)	
Internal barriers	**0.225, 0.053**	*−0.280, 0.153*	*−0.063, 0.037*	35.0%
	(0.121;0.329)***	*(−0.580;0.020)* ^†^	*(−0.136;0.010)* ^†^	
External barriers	**0.153, 0.053**	−0.237, 0.156	−0.036, 0.027	-
	(0.038;0.288)**	(−0.543;0.069)	(−0.089;0.017)	
**Peer interest in PA**				
*Potential mediators*				
Self-efficacy	**0.163,0.064**	0.029, 0.028	0.005, 0.005	-
	(0.038;0.288)*	(−0.026;0.084)	(−0.005;0.014)	
Internal barriers	**−0.037, 0.012**	**−0.311, 0.143**	*0.012, 0.006*	32.0%
	(−0.061;-0.012)**	(−0.591;-0.031)*	*(−0.001;0.024)* ^†^	
External barriers	**−0.029, 0.011**	*−0.268, 0.152*	0.008, 0.005	-
	(−0.051;-0.007)**	*(−0.566;0.030)* ^†^	(−0.003;0.018)	
**MVPA on weekend days**				
	**α (SE)**	**β (SE)**	**αβ (SE)**	**% mediated**
	**(95%CI)**	**(95%CI)**	**(95%CI)**	
**Parental co-participation in PA**				
*Potential mediators*				
Self-efficacy	0.042, 0.067	0.054, 0.056	0.002, 0.004	-
	(−0.089;0.173)	(−0.056;0.164)	(−0.006;0.011)	
Internal barriers	−0.009, 0.012	**−0.729, 0.306**	0.007, 0.009	-
	(−0.033;0.015)	(−1.329;-0.129)*	(−0.011;0.025)	
External barriers	−0.009, 0.012	**0.702, 0.324**	0.006, 0.009	-
	(−0.033;0.015)	(−1.337;-0.067)*	(−0.011;0.024)	
**Parental logistic support for PA**				
*Potential mediators*				
Self-efficacy	**0.175, 0.054**	0.025, 0.059	0.004, 0.010	-
	(0.069;0.281)**	(−0.091;0.141)	(−0.016;0.025)	
Internal barriers	**−0.045, 0.010**	*−0.585, 0.333*	0.026, 0.016	-
	(−0.065;-0.025)***	*(−1.238;0.068)* ^†^	(−0.005;0.058)	
External barriers	**−0.040, 0.010**	−0.545, 0.347	0.019, 0.013	-
	(−0.058;-0.021)***	(−1.225;0.135)	(−0.006;0.044)	
**Parental praise of PA**				
*Potential mediators*				
Self-efficacy	**0.103, 0.051**	0.039, 0.058	0.004, 0.006	-
	(0.003;0.203)*	(−0.075;0.153)	(−0.008;0.016)	
Internal barriers	**−0.031, 0.010**	*−0.585, 0.325*	0.018, 0.012	-
	(−0.051;-0.011)**	*(−1.238;0.068)* ^†^	(−0.005;0.041)	
External barriers	**−0.020, 0.009**	*−0.640, 0.330*	0.013, 0.009	-
	(−0.038;-0.002)*	*(−1.287;0.007)* ^†^	(−0.004;0.030)	

Analyses were adjusted for adolescents’ sex, maternal education, total accelerometer wear-time and days worn; β-path was adjusted for the independent variables; MVPA, moderate- to vigorous physical activity; SE, standard error; CI, confidence interval; ^**†**^p < 0.1 (indicated in italic); *p < 0.05 (indicated in bold); **p < 0.01 (indicated in bold); ***p < 0.001 (indicated in bold).

### Associations between potential mediators and MVPA (β-coefficients)

The β-path was significant or close to significant (and inverse) for perceived internal barriers and MVPA on weekdays and weekend days (Table 4). The β-path was inverse and close to significant for perceived external barriers and MVPA on weekdays and weekend days (all at p < 0.1 level). For all parent and peer variables, the β-path was not significantly associated between self-efficacy and MVPA on weekdays or weekend days.

### Mediating effect of barriers and self-efficacy on the associations between parental and peer variables and MVPA (αβ-coefficients)

Perceived internal barriers significantly mediated the association between parental logistic support and MVPA on weekdays (Table 4). Several close to significant mediating effects of perceived internal barriers were also found for the associations between parental control, parental concern and peer interest and MVPA on weekdays. Self-efficacy and perceived external barriers did not significantly mediate any association.

## Discussion

This study aimed to investigate associations between parental and peer variables and adolescents’ objectively measured mean MVPA on weekdays and weekend days and the possible mediating effect of perceived barriers and self-efficacy on these associations. The study found that, compared with the peer variables, a greater range of parental variables were associated with adolescents’ MVPA, and that these parenting variables influence adolescents’ MVPA on weekdays at least in part via perceived internal barriers such as lack of time and interest in PA. These findings have important practical implications for PA promotion among adolescents.

It has, however, been demonstrated in previous research that peers become more important as children grow and mature into adolescence [11]. A plausible explanation for our results might relate to the urban environment in Australia, where average distances to secondary schools are over 3 km [43] and adolescents’ independent mobility or the freedom to move around the neighbourhood without adult accompaniment is lower compared with other developed countries such as England [44]. Australian adolescents may therefore be more dependent on parental support to be physically active.

This study showed that parental logistic support was positively related to adolescents’ MVPA on weekdays and weekend days. Parental praise and parental co-participation were also both associated with adolescents’ MVPA on weekend days, although the association only approached significance. Parents should be made aware of the importance of their ongoing support of their child’s MVPA, even when their child becomes older and gains increased autonomy. This highlights the need for family-focused interventions to promote PA among adolescents, including strategies to increase parental support. This is especially the case for families who have reduced capacity to provide logistic support due, for example, to factors such as financial reasons, work patterns or time constraints. Davison and Jago [45] have suggested some strategies to facilitate the provision of logistic support, such as car-pooling to and from physical activities and organising social activities for parents while their child participates in sports activities. Other strategies to increase parental support include providing parents with suggestions on possible physical activities to do together with their child and encouraging sports clubs to inform and involve parents.

Parents play an important supportive role, however, parental control and concern about their adolescents’ PA levels were negatively related to MVPA on weekdays. This concurs with findings of another Australian study that reported an inverse association between parental restriction and weekday MVPA among adolescent girls [46] and suggests that the way in which parents support the activity of the adolescent is of great importance. This is consistent with studies investigating the impact of parenting styles and practices on adolescents’ food consumption where it has been shown that parental control is associated with a less favourable food intake [47, 48]. Thus, it would be important for parents to support the adolescent in order to increase MVPA without being too controlling. However, as our analyses are cross-sectional, it is also possible that parents might have a higher concern and more control regarding their child’s PA because their child is less physically active.

Regarding the peer variables, only peer interest in PA was related to adolescents’ MVPA on weekdays. It is possible that during the week, adolescents are more likely to be physically active during school hours (e.g. during recess or lunch break) or immediately after school if their friends are interested in PA. None of the peer variables were significantly related to adolescents’ MVPA on weekend days, which emphasises again the importance of parental variables as key correlates of adolescents’ PA. Additional research is needed to examine the impact of parental and peer variables on adolescents’ MVPA during specific periods (e.g. school hours vs. after school). According to the socio-ecological model [6] and the EnRG framework [7], these parental and peer factors can directly influence MVPA or can be mediated by personal factors. Almost all parental/peer variables were strongly associated with the personal variables, suggesting that personal factors can be shaped or reinforced by parents and peers [19].

In this study, only the perception of internal barriers was able to (partly) explain associations between parental/peer variables and MVPA on weekdays. The proportion of the associations explained by perceived internal barriers was high (up to 43%). These results suggest that if adolescents receive greater parental support (or if their closest friend is interested in PA), they may perceive fewer internal barriers to be physically active which may result in higher MVPA on weekdays. Although some results were close to significant, these findings suggest that parents may influence adolescents’ MVPA both directly and indirectly through the internalization of barriers. Previous research has identified the perception of internal barriers such as lack of time as an important correlate of adolescents’ PA [20] and this current study reinforces parents’ influence on this perception. Furthermore, it is possible that perceptions of internal barriers as a personal factors or cognition persist with age, such that adolescents who internalise fewer personal barriers during adolescence as a result of positive PA parenting practices may continue to be more physically active than others once the direct influence of their parents decreases.

Interventions should therefore focus on helping parents to support their child’s PA throughout youth to foster positive cognitions towards PA. Although our cross-sectional findings are in line with the theoretical EnRG-framework [7], longitudinal research and especially experimental research are needed to confirm this hypothesis and to make causal inferences, particularly given that reverse causality between MVPA and internal barriers is plausible, whereby higher MVPA levels may lead to fewer perceived internal barriers. Although several studies have cited self-efficacy as a consistent correlate of youth PA [8, 20–24], self-efficacy was not associated with adolescents’ MVPA on weekdays or weekend days in the current study, and as such was not a mediator of the associations between parental and peer variables and adolescents’ MVPA. Nevertheless, it has been argued that self-efficacy to overcome barriers might be more important for the initiation of PA, but that other forms of self-efficacy, such as self-regulatory efficacy, are more important for maintaining PA [8].

The absence of significant mediating effects for two of the three potential mediators suggests that the parental or peer variables may have a direct, automatic influence on MVPA. More likely, however, is that consistent with the EnRG framework [7], other personal variables not included in this study, such as perceived behavioural control, attitude and self-regulatory efficacy, might explain the association. Further research should include a range of other personal variables as mediators of associations between parental and peer factors and MVPA among adolescents.

A strength of this study is the effort to better understand the complexity of how parental and peer variables work together with personal factors to influence adolescents’ MVPA, which is currently an important target in behavioural research [22]. Another strength is the use of accelerometers to provide an objective view of adolescent’s MVPA levels. However, it could be argued whether or not data on one weekend day are sufficient to accurately estimate MVPA on weekend days. In the present study, 75% of adolescents had valid data on both weekend days. There are also other study limitations that need to be acknowledged. A first limitation is the use of single items to assess some of the parental and peer variables. Some items were combined to form particular constructs with high Cronbach’s alpha values being reported for all constructs except for parental control. A second limitation is the small sample size, partly due to the large attrition between T1 and T2. An important reason for the large attrition rate was that the study was not initially set up as a prospective cohort study. The small sample size did not allow for stratification by sex. It is possible that parental and peer influences differ [11] and that associations may operate through different personal factors, depending on sex. Also, this may affect the generalizability of the results and the ability to detect significant associations. Finally, this study was based on cross-sectional data and stronger study designs are needed to confirm the direction of associations between parental, peer, personal and PA variables among adolescents. Longitudinal research has the ability to make causal statements, but the strongest study design is experimental research to determine if an increase in parental support causes an increase in PA levels among adolescents in the intervention group compared to the control group and if this association is mediated by a decrease in the adolescents’ perception of internal barriers.

## Conclusions

Parents appear to play a more important role (e.g. providing logistic support) than peers regarding adolescents’ objectively measured MVPA on weekdays and weekend days. Parental support may also influence MVPA on weekdays by reducing adolescents’ perception of internal barriers such as lack of time and interest. Interventions aiming to increase adolescents’ MVPA should therefore include strategies directed to parents to help them support their adolescents’ PA. Longitudinal and experimental studies with a large sample are needed to confirm these findings and to investigate other potential mediators of the association between parental/peer factors and adolescents’ MVPA.

## Abbreviations

PA: Physical activity
MVPA: Moderate-to-vigorous physical activity
MET: Metabolic equivalent of task
SE: Standard error.
